# Supplementary material for: Pimavanserin and Parkinson’s Disease Psychosis: A Narrative Review
Source: Brain Sci. 2022 Sep 23;12(10):1286. doi: 10.3390/brainsci12101286 (PMC9599742; doi:10.3390/brainsci12101286)
Supplement: Supplementary file 1 [file brainsci-12-01286-s001.zip › brainsci-1894925-supplementary.pdf]

Pimavanserin And Parkinson's Disease Psychosis: A Narrative Review

SUPPLEMENTARY MATERIAL

Supplementary material S1 – Methodology
Supplementary materials S2 – Pharmacological
properties Supplementary material S3 – Pimavanserin
clinical trials Supplementary material S4 – 5-HT2A and
Diseases Supplementary Material S5 – Scale

Supplementary material S1 – Methodology

METHODS

Search Strategy

We searched six databases to locate the studies on pimavanserin published from 2006 to June 2022 in electronic form. Excerpta Medica (Embase), Google Scholar, Latin American & Caribbean Health Sciences Literature (Lilacs), Medline, Scientific Electronic Library Online (Scielo), and Science Direct were searched. Search terms were “pimavanserin, parkinson, psychosis” Publications in English and Spanish were included in the search.

Pimavanserin	"pimavanserin"[Supplementary Concept] OR "pimavanserin"[All Fields]	231
(pimavanserin) AND (parkinson)	("pimavanserin"[Supplementary Concept] OR "pimavanserin"[All Fields]) AND ("parkinson disease"[MeSH Terms] OR ("parkinson"[All Fields] AND "disease"[All Fields]) OR "parkinson disease"[All Fields] OR "parkinsons"[All Fields] OR "parkinson"[All Fields] OR "parkinson s"[All Fields] OR "parkinsonian disorders"[MeSH Terms] OR ("parkinsonian"[All Fields] AND "disorders"[All Fields]) OR "parkinsonian disorders"[All Fields] OR "parkinsonism"[All Fields] OR "parkinsonisms"[All Fields] OR "parkinsons s"[All Fields])	174
(pimavanserin) AND (psychosis)	("pimavanserin"[Supplementary Concept] OR "pimavanserin"[All Fields]) AND ("psychotic disorders"[MeSH Terms] OR ("psychotic"[All Fields] AND "disorders"[All Fields]) OR "psychotic disorders"[All Fields] OR "psychosis"[All Fields])	177

Figure – Number of publications on Pubmed/Medline throughout the years related to pimavanserin

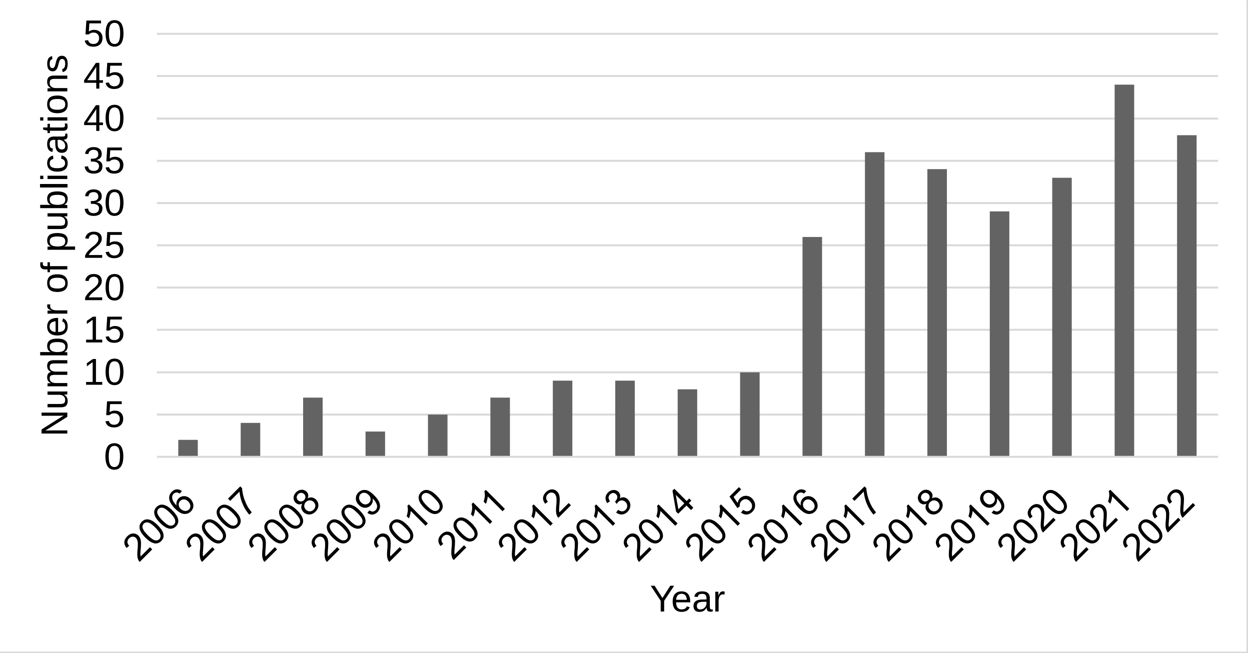

Supplementary materials S2 – Pharmacological properties

<table>
<tr><td colspan="2">Supplementary materials 2 – Pharmacological and physicochemical properties of pimavanserin</td></tr>
<tr><td>Skeletal formula</td><td>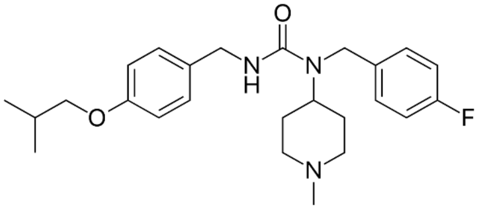
</td></tr>
<tr><td>SMILES</td><td>CC(C)COC1=CC=C(C=C1)CNC(=O)N(CC2=CC=C(C=C2)F)C3CCN(CC3)C</td></tr>
<tr><td>Other names</td><td>ACP-103; BVF-048</td></tr>
<tr><td>Trade Name</td><td>Nuplazid, Acadia Pharmaceuticals Inc</td></tr>
<tr><td>US FDA Approval</td><td>April 29, 2016</td></tr>
<tr><td>Indication</td><td>Treatment of hallucinations and delusions associated with Parkinson's disease psychosis</td></tr>
<tr><td>Dosage forms</td><td>Capsule 34mg; Tablet 10mg</td></tr>
<tr><td>Dosage adjustment</td><td>No need for dosage adjustment with renal or hepatic impairment. But cautiously use is advised.</td></tr>
<tr><td>Most common adverse effects (>1%)</td><td>Nausea, peripheral edema, confusional state, hallucinations, constipation, and gait disturbance</td></tr>
<tr><td>Black box warning</td><td>Elderly patients with dementia-related psychosis treated with antipsychotic drugs are at an increased risk of death.</td></tr>
<tr><td>Mechanism of action</td><td>Selective serotonin inverse agonist/antagonist activity preferentially targeting 5HT2Ar</td></tr>
<tr><td>Peak plasma</td><td>6 hours</td></tr>
<tr><td>Protein-bound</td><td>>95%</td></tr>
<tr><td>Volume distribution</td><td>2173L</td></tr>
<tr><td>Metabolism</td><td>Predominantly, CYP3A4 and CYP3A5. Others: CYP2J2, CYP2D6.</td></tr>
<tr><td>Half-life</td><td>57 hours (pimavanserin), 200 hours (active metabolite)</td></tr>
<tr><td>Excretion</td><td>55% unchanged in the urine</td></tr>
<tr><td>Formula</td><td>C25H34FN3O2</td></tr>
<tr><td>Molecular weight (150–500 g/mol)</td><td>427.55 g/mol</td></tr>
<tr><td>Fraction Csp3 (0.25–1.0)</td><td>0.48</td></tr>
<tr><td>Num. rotatable bonds (0-9)</td><td>10</td></tr>
<tr><td>Num. H-bond acceptors</td><td>4</td></tr>
<tr><td>Num. H-bond donors (≤5)</td><td>1</td></tr>
<tr><td>Topological polar surface area (20-130 Å²)</td><td>44.81 Å²</td></tr>
<tr><td>Consensus Log Po/w (0.7–5.0)</td><td>4.29</td></tr>
<tr><td>Log S (ESOL) (- 6–0)</td><td>-4.97</td></tr>
<tr><td>Class</td><td>Moderately soluble</td></tr>
<tr><td>Gastrointestinal absorption</td><td>High</td></tr>
<tr><td>Blood-brain-barrier permeant</td><td>Yes</td></tr>
<tr><td>Bioavailability score</td><td>0.55</td></tr>
<tr><td>Synthetic accessibility [1(very easy) – 10 (very difficult)]</td><td>3.02</td></tr>
<tr><td>Receptor selectivity of pimavanserin (Ki nM)</td><td><table>
<tr><td>5HT2A</td><td>5HT2B</td><td>5HT2C</td><td>5HT1A</td><td>H1</td><td>M1</td><td>M2</td><td>M3</td><td>M4</td><td>M5</td><td>D1</td><td>D2</td><td>D3</td><td>α1A</td><td>α2A</td></tr>
<tr><td>0.4</td><td>-</td><td>16</td><td>-</td><td>-</td><td>-</td><td>-</td><td>-</td><td>-</td><td>-</td><td>-</td><td>-</td><td>-</td><td>-</td><td>-</td></tr>
</table></td></tr>
<tr><td colspan="2">Physiochemical parameters computed with SwissADME. Daina A, Michielin O, Zoete V. SwissADME: a free web tool to evaluate pharmacokinetics, drug-likeness and medicinal chemistry friendliness of small molecules. Sci Rep 2017;7:42717. Hacksell U, Burstein ES, McFarland K, Mills RG, Williams H. On the discovery and development of pimavanserin: a novel drug candidate for Parkinson's psychosis. Neurochem Res 2014;39:2008-17.</td></tr>
</table>

Supplementary material S3 – Pimavanserin clinical trials

Summary of clinical trials related to pimavanserin				
Completion	Condition (or injury that is being studied)	CT identifier	n	Summary
-	PD psychosis	NCT02762591	-	Provide patients with PD psychosis access to pimavanserin until the product receives marketing approval from the FDA and is commercially available.
March 2007	Schizophrenia	NCT00361166	400	Efficacy, safety, and tolerability of the combination of pimavanserin with either haloperidol or risperidone.
November 2007	Levodopa-induced dyskinesias	NCT00086294	40	Efficacy of pimavanserin on PD symptoms and levodopa-induced dyskinesia.
July 2009	PD psychosis	NCT00477672	298	Safety and efficacy of pimavanserin in patients with PD psychosis.
December 2009	PD psychosis	NCT00658567	123	Safety and efficacy of pimavanserin in patients with PD psychosis.
November 2012	PD psychosis	NCT01174004	199	Safety and efficacy of pimavanserin in patients with PD psychosis.
May 2013	PD psychosis	NCT01518309	39	Long-term safety and tolerability in subjects with PD psychosis.
October 2016	Alzheimer's disease psychosis	NCT02035553	181	Safety and efficacy of pimavanserin in patients with Alzheimer's disease psychosis.
February 2018	Alzheimer's disease behavioral symptoms	NCT02992132	111	Safety and tolerability of pimavanserin in subjects with probable Alzheimer's disease who have symptoms of agitation and aggression.
May 2018	PD psychosis	NCT00550238	459	Long-term safety and tolerability of pimavanserin in subjects with PD psychosis.
October 2018	Major depressive disorder	NCT03018340	207	Efficacy of pimavanserin when given adjunctively to a selective serotonin reuptake inhibitor/ serotonin-norepinephrine reuptake inhibitor antidepressant as treatment of patients with major depressive disorder an inadequate response to antidepressant therapy.
February 2019	Alzheimer's disease behavioral symptoms	NCT03118947	79	Safety and tolerability of pimavanserin in subjects with probable Alzheimer's disease who have symptoms of agitation and aggression.
June 2019	Schizophrenia	NCT02970292	396	Efficacy and safety of adjunctive pimavanserin in the treatment of schizophrenia.
July 2019	PD depression	NCT03482882	47	Efficacy of pimavanserin for the treatment of depression in adults with PD.
October 2019	Dementia-related psychosis	NCT03325556	392	Efficacy of pimavanserin in preventing relapse of psychotic symptoms in subjects with dementia-related psychosis.
October 2019	Schizophrenia	NCT02970305	403	Efficacy and safety of adjunctive pimavanserin in the treatment of the negative symptoms of schizophrenia.
May 2020	Major depressive disorder	NCT03968159	298	To evaluate the efficacy and safety of adjunctive pimavanserin in subjects with a major depressive disorder who have an inadequate response to antidepressant therapy.
September 2020	PD	NCT04164758	11	Effects of pimavanserin and low-dose quetiapine in subjects with PD with neuropsychiatric symptoms.
February 2021	Tourette syndrome	NCT04794413	10	Efficacy of pimavanserin in Tourette syndrome. Also, the authors aimed to investigate the risk of tardive dyskinesia associated with pimavanserin.
February 2021	Major depressive disorder	NCT04000009	235	Safety and tolerability of long-term pimavanserin treatment in subjects with major depressive disorder and inadequate response to antidepressant therapy.

November 2021	Insomnia	NCT04188392	6	Efficacy of pimavanserin on sleep quality in veterans with post-traumatic stress disorder and insomnia.
May 2022	PD psychosis	NCT04292223	53	Efficacy of pimavanserin in PD psychosis.
May 2022	Neuropsychiatric symptoms	NCT03575052	784	Safety and tolerability of pimavanserin in adult and elderly subjects with neuropsychiatric symptoms related to neurodegenerative disease.
March 2023	Schizophrenia	NCT04531982	426	Efficacy and safety of adjunctive pimavanserin in managing negative symptoms of schizophrenia.
July 2023	Impulsivity in PD	NCT03947216	130	Efficacy of pimavanserin for the treatment of impulsive control disorder in PD.
August 2023	Neuropsychiatric symptoms	NCT03623321	597	Long-term safety and tolerability of pimavanserin in adult and elderly subjects with neuropsychiatric symptoms related to neurodegenerative disease.
December 2023	Insomnia	NCT04809116	20	Efficacy of pimavanserin on sleep quality in veterans with post-traumatic stress disorder and insomnia.
March 2024	Schizophrenia	NCT03121586	500	Long-term safety and tolerability of pimavanserin for adjunctive treatment in subjects with schizophrenia.
January 2025	Schizophrenia	NCT03994965	-	Translational proposal to test the clinical validity of the serotonin hypothesis of schizophrenia and to guide the development of operational, objective criteria for stratification of first-episode schizophrenia spectrum patients before antipsychotic treatment. More than 40 antipsychotics, including pimavanserin, will be studied.
August 2026	PD psychosis	NCT04373317	358	Safety and effectiveness of quetiapine and pimavanserin in PD psychosis.
May 2027	Insomnia	NCT05441280	60	Efficacy of pimavanserin on sleep quality in veterans with post-traumatic stress disorder and insomnia.

Data extracted from https://clinicaltrials.gov/. Search term: pimavanserin. Date: August 11th, 2022.
Completion: estimated study completion date; n: estimated enrollment (number of participants); PD: Parkinson's disease.

Supplementary material S4 – 5-HT2A and Diseases

Supplementary Material – 5-HT2A receptors and conditions related to the clinical trials of pimavanserin			
Condition	Mechanism	Reference	Clinical trials of pimavanserin
Impulsivity (IMP)	5-HT2A receptor antagonism decreases premature responding, which can lead to reduced impulsivity. Also, 5-HT2A receptor antagonism may unmask inhibitory effects of 5-HT at other 5-HT receptors.	Winstanley CA, Theobald DE, Dalley JW, Glennon JC, Robbins TW. 5-HT2A and 5-HT2C receptor antagonists have opposing effects on a measure of impulsivity: interactions with global 5-HT depletion. Psychopharmacology 2004;176:376-85.	NCT03947216
Insomnia (INS)	5-HT2A receptor antagonism enhances slow-wave sleep and delta power during non-rapid eye movement and decreases the number of awakenings without significant effect on rapid eye movement sleep.	Morairty SR, Hedley L, Flores J, Martin R, Kilduff TS. Selective 5HT2A and 5HT6 receptor antagonists promote sleep in rats. Sleep 2008;31:34-44.	NCT04188392<br>NCT04809116<br>NCT05441280
Depressive disorder (DD)	Decreased hippocampal 5-HT2A receptors result from depressive episodes. Decreased 5-HT2A receptors may not necessarily represent a detrimental change but could be part of a compensatory mechanism.	Mintun MA, Sheline YI, Moerlein SM, Vlassenko AG, Huang Y, Snyder AZ. Decreased hippocampal 5-HT2A receptor binding in major depressive disorder: in vivo measurement with [18F]altanserin positron emission tomography. Biol Psychiatry 2004;55:217-24.	NCT03018340<br>NCT03482882<br>NCT03968159<br>NCT04000009
Dyskinesia (DKN)	Post-mortem evidence of altered 5-HT2A receptor levels in levodopa-induced dyskinesia. 5-HT2A receptor agonism reduces dyskinesia in animal models. Selective 5-HT2A receptor blocking reduces dyskinesias over a specific limit. At high-doses 5-HT blockers could lead to a slight reduction in the effect of levodopa.	Kwan C, Frouni I, Bédard D, Nuara SG, Gourdon JC, Hamadjida A, et al. 5-HT2A blockade for dyskinesia and psychosis in Parkinson's disease: is there a limit to the efficacy of this approach? A study in the MPTP-lesioned marmoset and a literature mini-review. Exp Brain Res 2019;237:435-442.	NCT00086294
Schizophrenia (SCZ)	It was observed that patients with schizophrenia compared to healthy subjects have an abnormal binding potential for 5-HT2A receptors. These serotonin receptors control dopamine release in the nigrostriatal pathway.	Abdolmaleky HM, Faraone SV, Glatt SJ, Tsuang MT. Meta-analysis of association between the T102C polymorphism of the 5HT2a receptor gene and schizophrenia. Schizophr Res 2004;67:53-62.	NCT00361166<br>NCT02970292<br>NCT02970305<br>NCT04531982<br>NCT03121586<br>NCT03994965
Tourette syndrome (TS)	In TS, 5-HT2A receptors regulate postsynaptically to increase sensitivity for the remaining 5-HT. 5-HT2A receptor antagonists up-regulated 5-HT2A receptor decreasing 5-HT, which can further enhance dopamine regulation. Thus, the relationship between serotonin and dopamine receptors is probably associated with TS.	Wong DF, Brasić JR, Singer HS, Schretlen DJ, Kuwabara H, Zhou Y, et al. Mechanisms of dopaminergic and serotonergic neurotransmission in Tourette syndrome: clues from an in vivo neurochemistry study with PET. Neuropsychopharmacology 2008;33:1239-51.	NCT04794413

Supplementary Material S5 - Scales

Table – Scales performed on pimavanserin studies of Parkinson's disease psychosis					
Scale	Acronym	Number of items	Purpose	Considerations	Reference
Clinical Global Impression-Severity	CGI-S	6	Assessment of symptoms severity	Global assessment	Martínez-Martín P, Rojo-Abuin JM, Rodríguez-Violante M, Serrano-Dueñas M, Garretto N, Martínez-Castrillo JC, et al. Analysis of four scales for global severity evaluation in Parkinson's disease. NPJ Parkinsons Dis 2016;2:16007.
Clinical Global Impression-Improvement	CGI-I	6	Assessment of symptoms improvement	Global assessment	Bergman J, Lerner PP, Sokolik S, Lerner V, Kreinin A, Miodownik C. Successful use of escitalopram for the treatment of visual hallucinations in patients with Parkinson disease. Clinical Neuropharmacology 2017;40:246-50.
Epworth Sleepiness Scale	ESS	8	Daytime sleepiness	Unsuitable for screening for episodes of sudden sleep onset in patients with PD.	Kumar S, Bhatia M, Behari M. Excessive daytime sleepiness in Parkinson's disease as assessed by Epworth Sleepiness Scale (ESS). Sleep Med 2003;4:339-42.
Mini-Mental Status Examination	MMSE	11	Cognitive assessment	Easy and clear, most widely used screening tool for detecting dementia	Aarsland D, Muniz G, Matthews F. Nonlinear decline of mini-mental state examination in Parkinson's disease. Mov Disord 2011;26:334-7.
Neuropsychiatric Inventory	NPI	12	Cut-off psychosis	Efficient administration, separating symptom's frequency, some questions about specific psychotic phenomena	Pitton Rissardo J, Fornari Caprara AL. Parkinson's disease rating scales: a literature review. Ann Mov Disord 2020;3:3-22
Parkinson's Psychosis Rating Scale	PPRS	7	Psychosis assessment	Short, specifically to assess psychosis in PD. Fails to capture heterogeneous psychosis in PD.	Friedberg G, Zoldan J, Weizman A, Melamed E. Parkinson Psychosis Rating Scale: a practical instrument for grading psychosis in Parkinson's disease. Clin Neuropharmacol 1998;21:280-4.
Scale for the Assessment of Positive Symptoms - hallucinations and delusions subscales	SAPS-H+D	7 + 13	Psychosis assessment	Broad spectrum, including hallucinations and delusions. The scale was developed for schizophrenia.	Voss T, Bahr D, Cummings J, Mills R, Ravina B, Williams H. Performance of a shortened Scale for Assessment of Positive Symptoms for Parkinson's disease psychosis. Parkinsonism Relat Disord 2013;19:295-9.
Scale for the Assessment of Positive Symptoms – Parkinson's disease	SAPS-PD	9	Psychosis assessment	Developed by the assessment of pimavanserin efficacy	Schubmehl S, Sussman J. Perspective on Pimavanserin and the SAPS-PD: Novel Scale Development as a Means to FDA Approval. Am J Geriatr Psychiatry 2018;26:1007-1011.
Unified Parkinson's Disease Rating Scale Part II and Part III	UPDRS II/III	10 + 21	Activities in daily living and motor examination	Short, reliable, and valid, specifically for PD	Movement Disorder Society Task Force on Rating Scales for Parkinson's Disease. The Unified Parkinson's Disease Rating Scale (UPDRS): status and recommendations. Mov Disord 2003;18:738-50.
Zarit Burden Interview, Caregiver burden scale	CBS	22 items	Caregiver burden	Caregiver self-report measure	Giguère-Rancourt A, Plourde M, Racine E, Couture M, Langlois M, Dupré N, et al. Altered Theory of Mind in Parkinson's Disease and Impact on Caregivers: A Pilot Study. Can J Neurol Sci 2022;49:437-440.
